# Effect of Monoethylene Glycol on the Nucleation and Growth of Calcium Carbonate from Supersaturated Solutions in Microchannels of Varying Wettability

**DOI:** 10.1021/acs.langmuir.5c01363

**Published:** 2025-05-16

**Authors:** Andreas Tzachristas, Dimitra Kanellopoulou, John Parthenios, Petros G. Koutsoukos, Christakis Paraskeva, Varvara Sygouni

**Affiliations:** † Department of Chemical Engineering, 37795 University of Patras, Karatheodori 1, 26504 Patras, Greece; ‡ Institute of Chemical Engineering, Foundation for Research and Technology-Hellas, Stadiou str., Platani, 26504 Patras, Greece

## Abstract

The control of calcium carbonate formation is of high importance for a wide range of applications in the pharmaceutical industry and membrane processes as well as in the oil and gas industry. Herein, for the first time, the effect of monoethylene glycol (MEG) on the formation of calcium carbonate (CaCO_3_) crystals from supersaturated solutions flowing through microchannels (volume 0.36 mL) of varying wettability was investigated. The use of microdevices enabled the observation of the scaling phenomenon in the early stages. Solutions supersaturated with respect to calcite, containing MEG (10, 20, and 30% v/v), were injected into the microchannel under a constant total flow rate and under laminar flow conditions (Re = 0.052). The growth of calcium carbonate crystals was monitored by video recording. The effect of the wettability on crystal formation was tested using glass and silane-coated microchips. The microchannel walls were wet and neutral-wet by the supersaturated solutions with a low MEG concentration. In the presence of a high MEG concentration in the supersaturated solutions, the walls of both types of microchips were neutral-wet. The results showed that the addition of MEG at a concentration of 10% v/v in the supersaturated solutions decreased the time of observation of the first crystal, favored secondary nucleation, and in general decreased the crystal growth rates. Raman spectroscopy identified the formation of aragonite in most cases, while as SR values increased, the formation of aragonite aggregates was favored. Further increases of the MEG concentration in the supersaturated solutions to 20% v/v, at low supersaturation ratio (SR) values, favored the formation of amorphous calcium carbonate (ACC) while at higher SR values aragonite crystals and aragonite aggregates formed. Further increases in the MEG concentration in the supersaturated solutions up to 30% v/v completely inhibited the nucleation and crystal growth of calcium carbonate.

## Graphical abstract

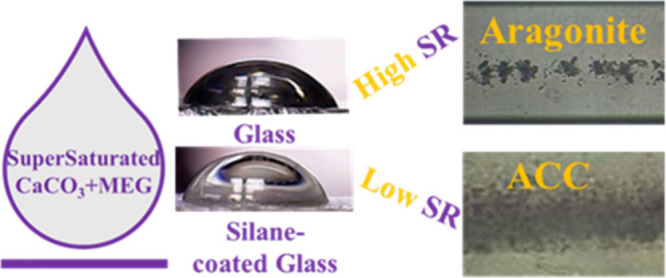

## Introduction

Calcium carbonate’s polymorphs, from the most stable, calcite, to the unstable, vaterite, aragonite, calcium carbonate hexahydrate (CaCO_3_·6H_2_O, ikaite), and amorphous calcium carbonate (ACC) in increasing order of solubility, have been of high interest to the scientific community for many years.^1–3^ Calcium carbonate formation under various conditions of temperature, pH, and in the presence of additives has been investigated in various applications.^4–6^ In pharmaceutics, CaCO_3_ is a low-cost biocompatible drug carrier and may be used for the improvement of controlled drug release.^7^ Vaterite particles are characterized by their hexagonal structure and high loading volume. Vaterite for drug delivery for photodynamic therapy was prepared using a sodium poly­(styrenesulfonate) route.^8^ Vaterite particles were synthesized with the addition of hydroxypropyl methyl cellulose and sodium dodecyl sulfate and were found to have a convex-disk shape.^9^ Moreover, they were loaded with an anticancer drug and were found to be satisfying carriers based on their loading capability and the drug release profiles.^9^ Apart from the pharmaceutical industry, the formation of CaCO_3_ is of high importance in diverse areas such as the gas and oil production industry, carbon capture utilization and storage, and membrane processes. More specifically, during multiphase flow processes encountered in these and related processes, the formation of sparingly soluble salts like CaCO_3_ damages the equipment and decreases the efficiency of several processes. Thus, control of calcium carbonate polymorphs formation is of high interest for several applications.^10,11^

In the gas and oil industry, a widely used green inhibitor of the formation of crystalline gas hydrates is monoethylene glycol (MEG).^12,13^ The presence of MEG, however, in the formation water containing high concentrations of dissolved minerals, may trigger the formation of scale deposits of sparingly soluble salts.^14,15^ MEG was found to delay the formation of calcite and vaterite while it did not inhibit the formation of aragonite.^16^ Calcium carbonate was precipitated by mixing solutions containing calcium and bubbling CO_2_ under stirring. Induction times preceded calcium carbonate precipitation, which increased as the MEG concentration in the supersaturated solutions increased. The effect was more pronounced at low temperatures.^17^ In the presence of MEG, the interfacial water/solid tension was reduced, the nucleation rates were higher, and the crystal growth rates decreased.^17^ This was probably an indication that the presence of MEG in the supersaturated solutions acted only as a crystal growth inhibitor. The significant role of MEG in the kinetics of calcium carbonate formation of less stable polymorphs (i.e., vaterite and aragonite in batch experiments of calcium carbonate precipitation) has been reported.^18^ More specifically, it was observed that the presence of MEG with concentrations from 20 to 30% v/v and higher calcium carbonate supersaturated solutions with low SR values inhibited nucleation of the solid phase. In solutions of higher SR values, nucleation was not inhibited and diffusion on the surface of the nucleated crystals was the predominant mechanism for crystal growth.^18^ The rate of precipitation of calcium carbonate was reported to increase with increasing MEG concentration even though the effective SR values were lower.^18^ Calcium carbonate precipitation in stainless steel tubes under dynamic and static conditions, at 72 °C in the presence of MEG (0–61% m/m), showed that increasing MEG concentration at high salinities increased the time for calcium carbonate scale formation.^19^ At high SR values, increasing MEG concentration increased the viscosity of the aqueous supersaturated solutions, resulting in reduced crystal agglomeration and deposition on the walls. Increasing SR and MEG concentrations in the supersaturated solutions resulted in higher nucleation rates but lower crystal growth rates of calcium carbonate.^19^

The apparent linear rate constant of the precipitation of calcium carbonate in the presence of MEG and at temperatures between 40 and 70 °C decreased from 0.52 to 0.11 nm/s upon increasing MEG concentration from 0 to 65 wt %.^20^ Calcium carbonate precipitation upon mixing a sodium carbonate solution with calcium chloride under stirring in the presence of PEG polymers showed that calcite and vaterite are the predominant polymorphs. The formation of vaterite crystals was not favored at higher polyethylene glycol (PEG) concentrations. At higher PEG concentrations, crystals of smaller size formed, possibly because of the adsorption of ethylene oxide groups on the ACC crystallites, the growth of which to larger size was inhibited.^21^ The kinetics of vaterite growth at high temperatures was found to depend on the MEG concentration.^22,23^ Calcium carbonate formation during the flow of calcium carbonate supersaturated solutions containing MEG (10% v/v), in beds packed with sand, showed that the presence of MEG favored the formation of calcium carbonate crystals along the beds. Moreover, the development of intragranular crystals enabled the consolidation of the grains, largely preserving the permeability of the sand beds.^24^ The precipitated crystals consisted of calcite because of the high total calcium concentration in the supersaturated solutions (i.e., high SR values) and the long duration of the precipitation process (exceeding 4 days).^24^ It is therefore obvious that although many research studies have focused on the effect of MEG on CaCO_3_ formation and growth, the phenomenon in its initial stages has not yet been described adequately, information on prevailing mechanisms in the initial stages of the precipitation process is still needed. Microfluidic experiments have an advantage on visualizing phenomena in their early stages compared to batch experiments where larger volumes are used.^25–27^ To this direction, in this work, we present the findings from microfluidic experiments in which the formation of calcium carbonate crystallites from supersaturated solutions in the presence of MEG was monitored through direct video recording. A similar investigation of CaCO_3_ precipitation in the absence of MEG in neutral-wet and water-wet (hydrophilic) microchips for various SR values showed that for laminar Stokes flow, under relatively low flow rates, hydrophobicity accelerated crystal nucleation. Moreover, it was shown that the formation of calcium carbonate metastable polymorphs (vaterite and aragonite) was favored in the case of neutral-wet microchips.^25^ This effect was attributed to local heterogeneities of supersaturation values due to the higher contact angle of the solution with the microchannel walls.^25,28^ The present work is focused on the investigation of the presence of MEG in the calcium carbonate supersaturated solutions in CaCO_3_ crystal formation and growth and is an extension of our earlier work on the precipitation of calcium carbonate in batch stirred reactors.^18^ In this study, the effect of the wettability of the walls of the microchip containing the supersaturated solutions on the formation of calcium carbonate crystals, in the presence of MEG, is investigated. The scale down of the calcium carbonate precipitation from batch reactors to microchips involved very small volumes of the supersaturated solutions under flow conditions. The precipitation of calcium carbonate was investigated using supersaturated solutions (SR values of 10.5 and 30.2) containing MEG (10 and 20% v/v) flowing in glass and silane-coated microchips. The MEG concentration at 30% v/v showed the complete inhibition of crystal nucleation, and this concentration was not tested further. This phenomenon at 30% v/v MEG was also observed in previous studies.^17,18^ More specifically, the induction times were increased with increasing MEG concentration at values higher than 30% v/v, and this effect was more significant at lower temperatures (∼25 °C).^18^ The presence of MEG is expected to affect not only the solvent–ion but also the solvent–microchannel wall interactions, possibly modifying the kinetics of calcium carbonate precipitation and the polymorphic composition of the mineral precipitating out.

In the present study, microchannels are used for the direct optical recording of the initial stages of the precipitation process during the flow of supersaturated solutions with the addition of MEG. The technologically advanced Y-junction microchips and the use of micropumps allow solution mixing inside the microchannel and the observation of phenomena in their early stages. The use of microvolumes and the short residence time of liquids allow us to neglect the supersaturation ratio’s gradient along the microchip and the gravity effects. This experimental work provides information on the effect of the MEG presence on CaCO_3_ precipitation in microchannels of different wettabilities under continuous laminar flow conditions where the dynamics differ from batch experimental conditions. The observations obtained from the continuous flow experiments focus on heterogeneous nucleation due to the existence of the microchannel’s wall surface. The obtained crystal growth rates and the detected stabilized polymorphs provide new insights concerning the inhibition mechanism of MEG’s presence in the supersaturated solutions under the specific conditions. This information may be used in future studies to simulate the calcium carbonate formation in channels.

## Experimental Section

### Supersaturated Solutions

The driving force for the formation of calcium carbonate precipitation from supersaturated solutions depends on the supersaturation ratio (SR_*x*_), with respect to polymorph phase x (x corresponds to vaterite, aragonite, and calcite) defined according to eq 1

1 $${SR}_{x}=\frac{\left(Ca^{2+}\right)\left(C{O_{3}}^{2-}\right)}{K_{s,x}^{0}}$$

where parentheses denote the activities of the respective ions and K_s,x_^0^ is the thermodynamic solubility product of polymorph x. Based on the classical nucleation theory, in the absence of foreign substances (organic substances, nanoparticles, seeds, etc.) existing in the fluid system, supersaturation is the driving force for nucleation which is called homogeneous.^2^ In other systems where seeds, foreign particles, or surface walls are in direct contact with the supersaturated solutions, the nucleation process is heterogeneous. In this study, in the presence of the organic phase (MEG) in the supersaturated solution and using solutions which were prepared in the laboratory open air, and since the process taking place inside the microchannel is heterogeneous precipitation.^2^

All supersaturated solutions were prepared from stock calcium chloride (CaCl_2_) and sodium bicarbonate (NaHCO_3_) solutions prepared from the respective crystalline solids (Merck, reagent grade). In all solutions triply distilled water was used and they were filtered through membrane filters (Sartopore 0.2 μm). Calcium chloride stock solutions were standardized with atomic absorption spectroscopy (AAS, PerkinElmer, AAnalyst 300). Sodium bicarbonate stock solutions were prepared from the respective crystalline solid. These solutions were prepared fresh every day. Sodium chloride stock solutions were prepared from the crystalline solid (Merck, reagent grade) and dried overnight (65^ο^C). Equal volumes of calcium chloride and sodium bicarbonate solutions were prepared by dilution from the respective stock solutions. The ionic strength of all solutions was adjusted to 0.15 M, with NaCl from the stock solution. MEG (Merck, purity >99.5%) was directly diluted in the supersaturated solutions to achieve concentrations equal to 10, 20, and 30% v/v. The pH values were measured in the aqueous solutions (pH = 7.91 for SR = 10.5 and pH = 7.86 for SR = 30.2). SR values were calculated using appropriate software.^29^ The SR values in the presence of MEG were calculated (and adjusted accordingly) from earlier measurements on the MEG-calcium carbonate solutions of the partial molar volumes and viscosity measurements over a broad range of MEG concentrations.^18^ Low SR values were selected to reduce the number of crystals formed, to avoid secondary nucleation, and to obtain higher accuracy in crystal growth rates. Next, the two solutions were injected into the microchip and were mixed under continuous flow conditions at room temperature. The room temperature was retained at 25 °C; however, this is not a crucial parameter. The value of the activation energy depends mainly on the underlying mechanism. For mass transport control (bulk diffusion), the apparent activation energy is <20 kJ/mol while for surface diffusion-controlled processes (most of the related literature in batch-type reactors is consistent with this mechanism) the apparent activation energy is on the order of ca. 60 kJ/mol.^30^

### Experimental Setup and Microchannel

The precipitation of calcium carbonate from supersaturated solutions was investigated using the experimental setup illustrated in Figure 1.

**Figure 1:**
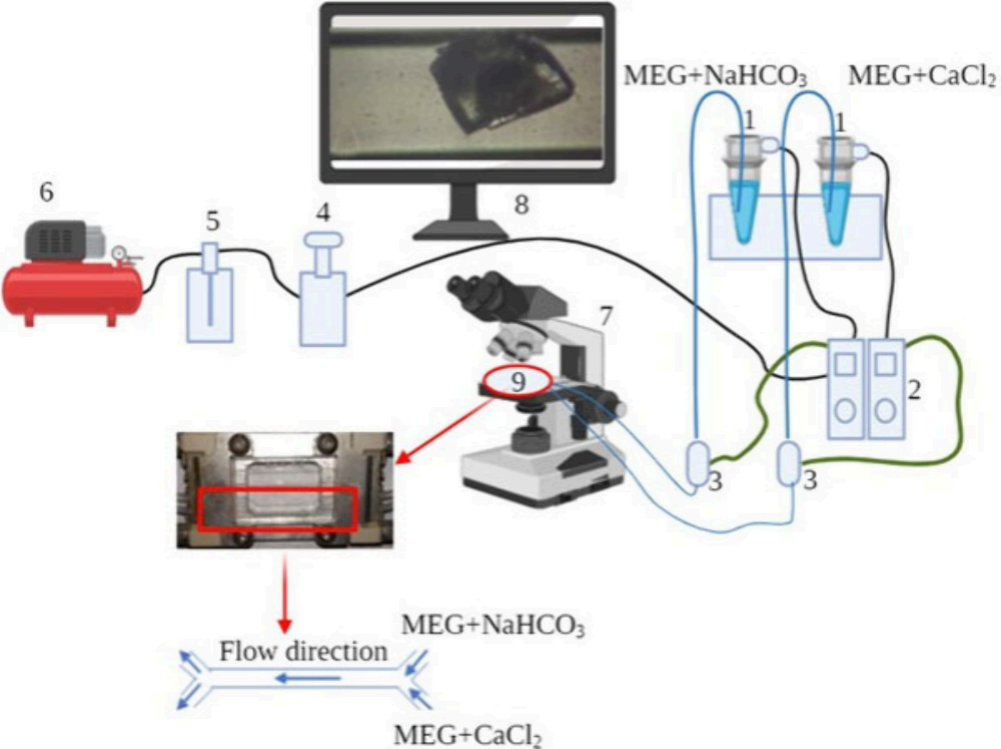
Experimental setup: (1) falcon tubes equipped with massive aluminum caps for pressurization containing the solutions, (2) flow controller (LineUP Flow EZ, Fluigent Smart Microfluidics 1000mbar), (3) flow units (0–500 μL/min), (4) regulator, (5) air drier, (6) air pump (1 bar, 230 V, 50 Hz), (7) Zeiss microscope and digital video camera (Axis 223 M), (8) computer, and (9) microchip.

A Zeis microscope (microscope objective Olympus E A10) with a digital programmed video camera (Axis 223 M) was used to monitor the crystals along the microchannel. Snap shots along the microchannel were taken every 5 min after the observation of the first crystal. The threshold size for the observation of crystals was at least 4–5 μm. Smaller crystal sizes could not be detected by the optical microscope in the experimental setup. The solutions were placed in falcon tubes equipped with massive aluminum caps for pressurization, which did not permit the interaction with the CO_2_ of the atmosphere. The fluids were injected into the microchannel using the system of microfluidic devices (LineUP Flow EZ, Fluigent Smart Microfluidics 1000 mbar) (Figure 1).^25,31^ The crystallization of calcium carbonate took place in Y-junction microchannels of 0.2 μL volume between the Y-junctions (Dolomite, Royston, U.K.). The length of the channel between the Y-junctions was 1.5 cm, while the width was 205 μm and the height was equal to 100 μm. The cross section of the microchannels was u-type. The microchips had two inlets and two outlets allowing for the in situ mixing of solutions during the flow of the solutions, making up the final supersaturated solution with respect to calcium carbonate.^25^ The experiments were performed under laminar Stokes flow conditions (total flow rate q_t_ = 0.5 μL/min, u = 2.53 × 10^–4^ m/s, Re = 0.052) to allow approximately 98% homogeneous mixing of the solutions at a distance ∼6 mm from the mixing point. Experimental tests under various flow rates under laminar flow conditions showed that at the specific flow rate homogeneous mixing is effective over 60% of the length of the microchip between the Y-junctions, enabling the observation of crystal formation over a more extended channel length. Previous studies showed that surface roughness accelerated the nucleation process.^32^ The low surface roughness of the microchannel’s walls (5 nm) was an advantage of this type of microchip since the contribution significance of this parameter was minimized.^32^ The microchips used in the present work were constructed of B 270-type glass, while the microchannels with additional coating of silane groups were also used to investigate the effect of the wettability of the wall’s surface on the precipitation process of calcium carbonate inside the microchannels. Sampling and measurements at the outlet of the microchannel were not possible due to the very small used volumes. The use of microchips as precipitation reactors of calcium carbonate offered the advantage for obtaining information on crystal growth mechanisms. The formation and growth of crystals under continuous flow in the confined space between the Y-junctions (0.2 μL) are important because, in combination with the flow rate, they affect the residence time of the supersaturated solutions in the microchip. During the precipitation experiments, the supersaturation ratio decreases along the channel due to the consumption of calcium ions in the crystal formation. However, the supersaturation ratio gradient is practically negligible for small volumes and short residence times.^25^ The time of fluid residence in the microchannel for the flow rate used was 0.4 min. As the supersaturated solutions were prepared in situ inside the microchannel under a continuous flow of the stock calcium chloride and sodium bicarbonate solutions, the first crystal embryos formed and grew by the addition of growth units from the surrounding supersaturated solution. Crystal growth and evolution were therefore directly monitored at sustained supersaturation. The experiments were performed in duplicate. The combination of Raman spectroscopy was explained in detail in previous studies,^25,33^ and specific sampling of the present study and the morphological characteristics of the crystals allowed the extraction of the conclusions concerning the formed crystal polymorphs.

### Properties of Fluids

The static contact angles of the test solutions were measured on glass and on silane-coated glass (Table 1). For contact angle values below 60 °C, the fluid system used is usually considered to wet a solid surface. When measured contact angles are between 60 and 90 °C, the wettability is considered neutral or intermediate. For contact angles exceeding 90 °C, it is considered that the fluid system does not wet the surface.^34^ The presence of MEG in the solutions inside the microchannel increased the aqueous solution–glass contact angle. The increase in the contact angle was more significant for the solution with the higher MEG concentration (20% v/v). Concerning the contact angle of the test solutions with the silane-coated glass surface, there was a slight decrease, but the wettability remained neutral.^34^ The viscosity and density of the test solutions did not change significantly with the addition of MEG. MEG’s presence decreased the surface tension (air/solution) of the test solutions. In the case of the CaCO_3_ saturated solution with 20% v/v MEG, there was a small difference between the contact angles for the two microchips. Characteristic properties of calcium carbonate solutions saturated with respect to calcite are summarized in Table 1.

**Table 1:** Properties of Aqueous Solutions of Calcium Carbonate Saturated with Respect to Calcite: Contact Angle (θ) of Solution/Glass, Solution/Silane-Coated Glass, Viscosity, and Density

Fluid	θ on glass surface (deg)	θ on silane-coated glass surface (deg)	Viscosity (mPa s)	Density (g/mL)
CaCO_3_ saturated solution	0	85	1	1
CaCO_3_ saturated solution (10% v/v MEG)	∼25	75	5	1.012
CaCO_3_ saturated solution (20% v/v MEG)	63	71.5	7	1.028

Surface tension measurements of solutions used in the experiments were measured using a K20 force tensiometer (Krüss GmbH) (Table 2). As can be seen, the measured values of the separate supersaturated solutions (CaCl_2_ and NaHCO_3_) which were injected into the microchip as well as the supersaturated CaCO_3_ solutions formed during their flow were characterized by surface tension values lower than the corresponding value for deionized water. All prepared solutions contained appropriate NaCl quantities for the ionic strength’s value adjustment at IS = 0.15 M and were vacuum filtered before the measurement of surface tension. Although most published studies show that the addition of electrolytes increases surface tension values,^35,36^ it is reported that, in the low electrolyte concentration range, the surface tension decreases due to the Jones-Ray effect and not due to the surfactant’s presence.^37,38^ The surface tension values for the corresponding solutions upon addition of MEG are generally in agreement with reported values in the literature, taking into consideration relatively small differences which could be attributed to the composition of the supersaturated solutions.^39^

**Table 2:** Surface Tension Measurements of Solutions Used in the Experiments

Solution	Surface tension (mN/m)
Deionized water	72.16 ± 0.03
NaHCO_3_ (25 mM, IS = 0.15 M)	60.28 ± 0.24
CaCl_2_ (25 mM, IS = 0.15 M)	63.62 ± 0.24
CaCO_3_ (mix of NaHCO_3_ 25 mM and CaCl_2_ 25 mM, SR = 30.2, 0.15 M)	61.13 ± 0.18
CaCO_3_ (SR 30.2, IS = 0.15 M) (10% v/v MEG)	61.01 ± 0.18
CaCO_3_ (SR 30.2, IS = 0.15 M) (20% v/v MEG)	55.35 ± 0.33
NaHCO_3_ (13 mM, IS = 0.15 M)	63.91 ± 0.11
CaCl_2_ (13 mM, IS = 0.15 M)	67.68 ± 0.05
CaCO_3_ (mix of NaHCO_3_ 13 mM and CaCl_2_ 13 mM, SR = 10.5, IS = 0.15 M)	67.39 ± 0.02
CaCO_3_ (SR 10.5, IS = 0.15 M) (10% v/v MEG)	66.77 ± 0.01
CaCO_3_ (SR 10.5, IS = 0.15 M) (20% v/v MEG)	63.23 ± 0.03

## Results and Discussion

### CaCO_3_ Crystal Nucleation and Growth in Microchannels in the Presence of 10% v/v MEG

Crystal growth of CaCO_3_ during the flow of supersaturated solutions in glass and silane-coated microchips was investigated for two different SR values (SR = 10.5 and 30.2) containing MEG (10% v/v). All solutions in the present work were supersaturated with respect to all calcium carbonate polymorphs. Metastable phases, however, may be kinetically stabilized for longer or shorter times before converting to the thermodynamically most stable calcite.

In the case of the glass microchannel and at SR = 10.5, the first crystal was observed past 5.5 h from the start of the flow of supersaturated fluid. The rhombohedral shape of the crystal suggested the formation of calcite (Figure 2(a)).^18,25^ In the absence of MEG under similar experimental conditions in the glass microchannel, the first calcite crystal was detected 34 h past the beginning of the injection of supersaturated solution^25^ (i.e., the presence of MEG accelerated the crystal nucleation). Induction time preceding the formation of crystals in the supersaturated solutions, according to the classical nucleation theory (CNT), reflects the incubation time needed for the formation of the nucleus of critical size.^40^ The reduced time of observation of the first crystal agrees with other study results where MEG triggered crystal formation.^14,15^ By increasing the supersaturation ratio SR to 30.2 in the glass microchip in the presence of MEG (10% v/v), the time of the first observed crystal was reduced to 1.0 h of fluid flow (Figure 2(b)). In the absence of MEG, 24 h of fluid flow lapsed before the observation of the first crystal in the microchannel reactor.^25^ This result was also in agreement with the prediction of CNT, according to which increasing the thermodynamic driving force for the formation of a solid phase from supersaturated solutions causes a sharp decrease in the respective induction times preceding the onset of crystal formation.^40^

**Figure 2:**
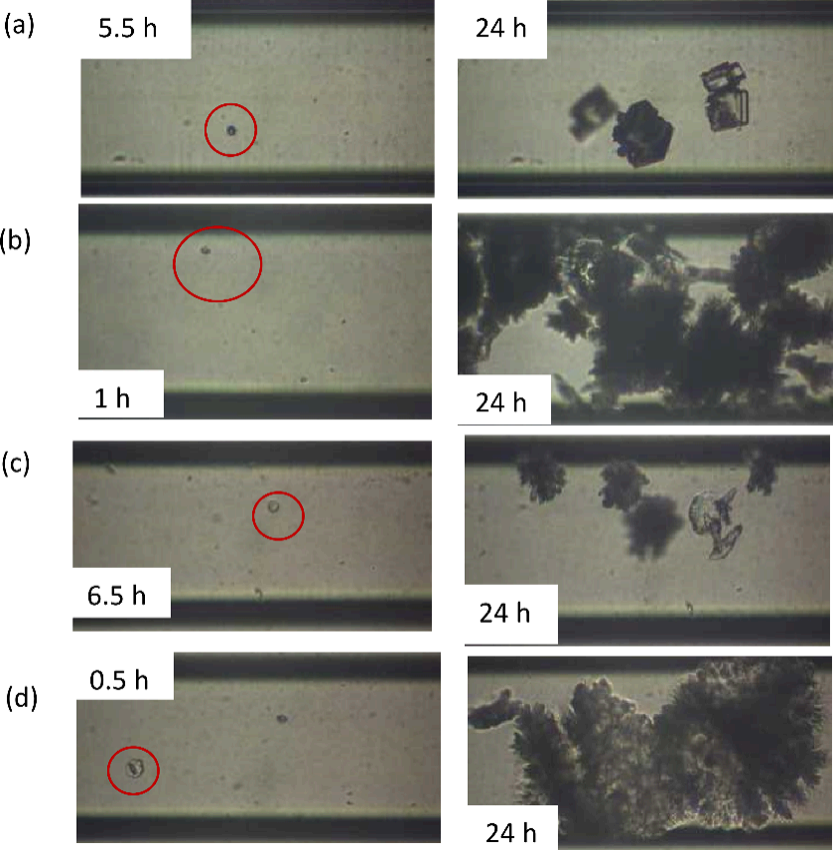
Snapshots of the first detected CaCO_3_ crystal in the presence of MEG (10% v/v) at (a) SR = 10.5 in the glass microchannel, (b) SR = 30.2 in the glass microchannel, (c) SR = 10.5 in the silane-coated microchannel, and (d) SR = 30.2 in the silane-coated microchannel.

In the case of the silane-coated microchannel (i.e., the microchannel was neutral-wet by the supersaturated solution) at SR = 10.5 and at MEG 10% v/v, the first crystal was observed after 6.5 h of fluid flow (Figure 2(c)) while in the absence of MEG and under similar experimental conditions ACC and aragonite crystals were formed along the entire length of the microchannel.^25^ At a higher SR value (30.2) and in the presence of MEG (10% v/v) in the supersaturated solutions, the time lapsed for the observation of the first crystal was even shorter (0.5 h) (Figure 2(d)) in comparison with the respective time in the absence of MEG (3 h). The prismatic shape of the crystals, elongated along the *c* axis, corresponded to the less stable polymorph aragonite. Aggregates of aragonite crystallites formed most likely because of the high SR value of the corresponding supersaturated solutions. The differences in contact angles of the supersaturated solutions with the glass and silane-coated surfaces were reflected in the shorter times of the appearance of the first crystal in the silane-coated microchips due to local inhomogeneities in SR near the neutral water-wet.^25,28,41^ The presence of MEG lowered the surface tension of the supersaturated solutions (Table 2); therefore, the Gibbs free energy for crystal nucleation and growth decreased, making both wettability conditions in the presence of MEG, nucleation, and the growth of calcium carbonate more favorable.^24,25^

During the flow of supersaturated solutions (SR = 10.5) containing MEG 10% v/v in the glass microchannel, at the areas near the entrance, small crystals resembling aragonite were formed, and at longer distances, calcite crystals were precipitated. In the areas closer to the entry point where the mixing of the supersaturated fluid was not fully homogeneous, less stable polymorphs are favored, while calcite crystals formed in areas at longer distances where supersaturated solution mixing was homogeneous (Figure 3).^25^ In the areas near the entrance, the crystal formation activity was higher on one side of the channel, possibly due to the higher concentration of [CO_3_^–2^] and the differences in diffusion coefficient values of the involved ions. In the absence of MEG, only a few calcite crystals were detected in the same type of microchannel reactor (i.e., MEG not only accelerated crystal nucleation but also increased the number of nucleation sites and thus the number of crystals).^25^

**Figure 3:**
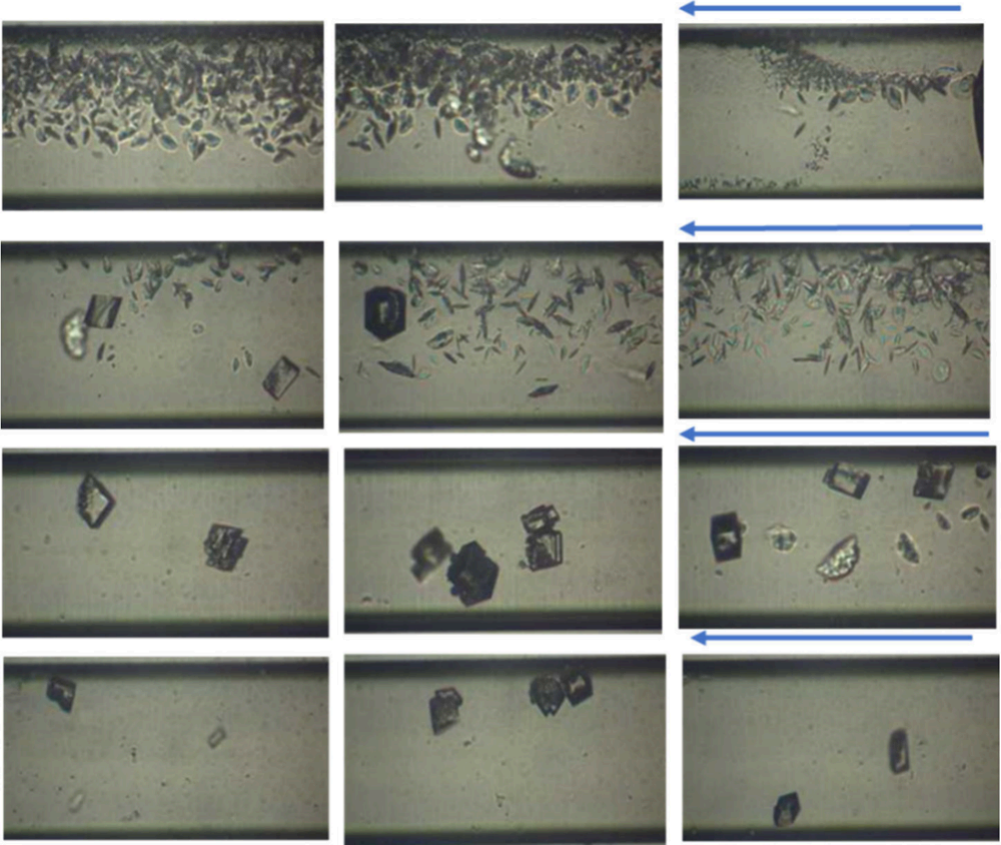
CaCO_3_ crystal growth along the glass microchannel in the presence of MEG 10% v/v at SR= 10.5 (24 h past the initiation of solution injection). Arrows show the flow direction.

By increasing SR to 30.2 in the presence of MEG (10% v/v), the formation and stabilization of aragonite aggregates were favored along the glass microchannel (Figure S1) while in the areas near the entrance the aggregates do not have a morphology which refers to a specific polymorph. Obviously, the increase in the apparent SR in the presence of MEG resulted in the formation of a less stable polymorph (i.e., aragonite).

In the silane-coated microchannel, during the injection of supersaturated solutions at an SR equal to 10.5, the presence of MEG resulted in the formation of aragonite aggregates along the microchannel (Figure S2). However, it should be noted that in the absence of MEG in the silane-coated microchannel, under similar experimental conditions, aragonite prevailed.^25^ The presence of MEG therefore increased nucleation sites, resulting in aggregate formation. At a higher SR value (30.2) and in the presence of MEG (10% v/v), a few crystals precipitated in the silane-coated microchannel (Figure 4). The formation of aragonite aggregates resulted in microchip clogging and calcite formation in areas near the entrance of the microchip.

**Figure 4:**
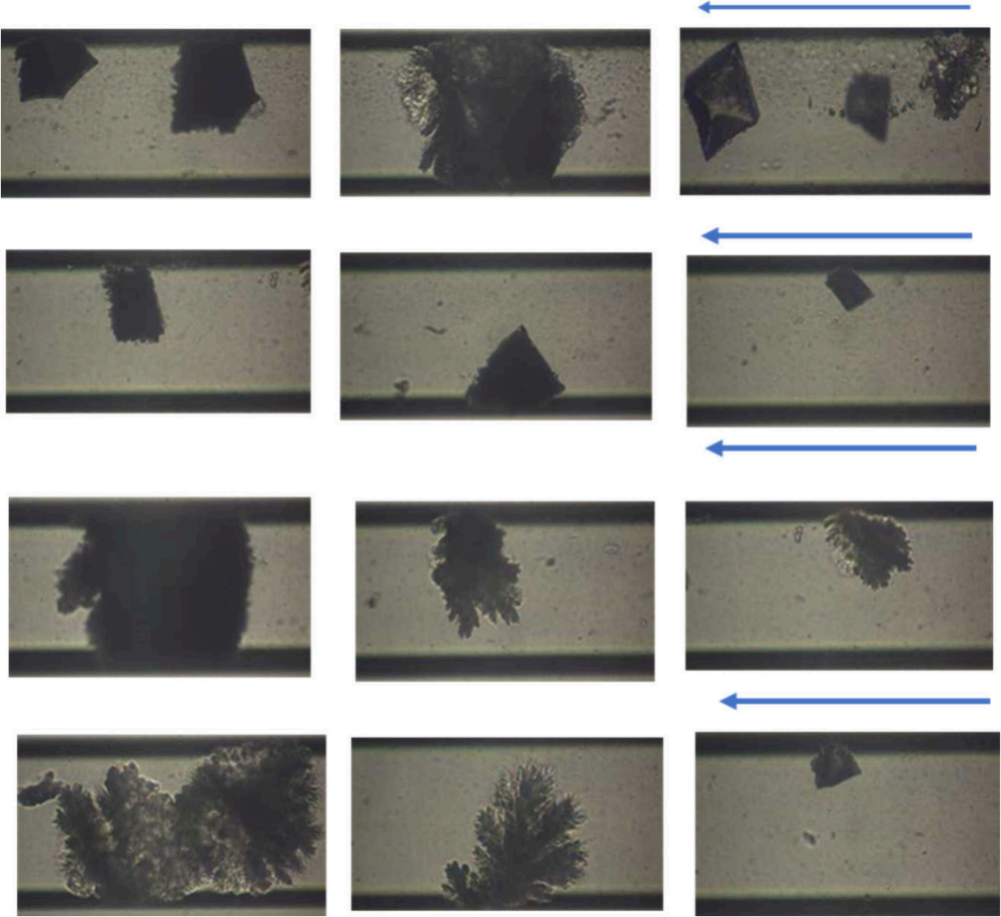
CaCO_3_ crystal growth along the silane-coated microchannel in the presence of MEG 10% v/v at SR = 30.2 (24 h past the initiation of solution injection). Arrows show the flow direction.

### CaCO_3_ Crystal Nucleation and Growth in Microchannels in the Presence of 20% v/v MEG

Increasing the concentration of MEG in the supersaturated solutions from 10 to 20% v/v affected the contact angles of the supersaturated solutions with the microchannel walls. There was only a small difference in the contact angles for the two types of microchips (Table 1). For both glass and silane-coated microchips, wettability was almost neutral with a slightly lower contact angle for the glass surface. In the glass microchannel at SR = 10.5, 1 h past the initiation of solutions flow , only ACC was formed (Figure 5(a)). At a higher SR value (30.2) and in the presence of MEG 20% v/v in the glass microchannel, the first crystals were observed 0.16 h past the start of the injection of the supersaturated solutions (Figure 5(b)). The outline of the shape of the precipitated crystals suggested the formation of calcite and aragonite, with the latter more than the particulate precipitate in the microchannel.

**Figure 5:**
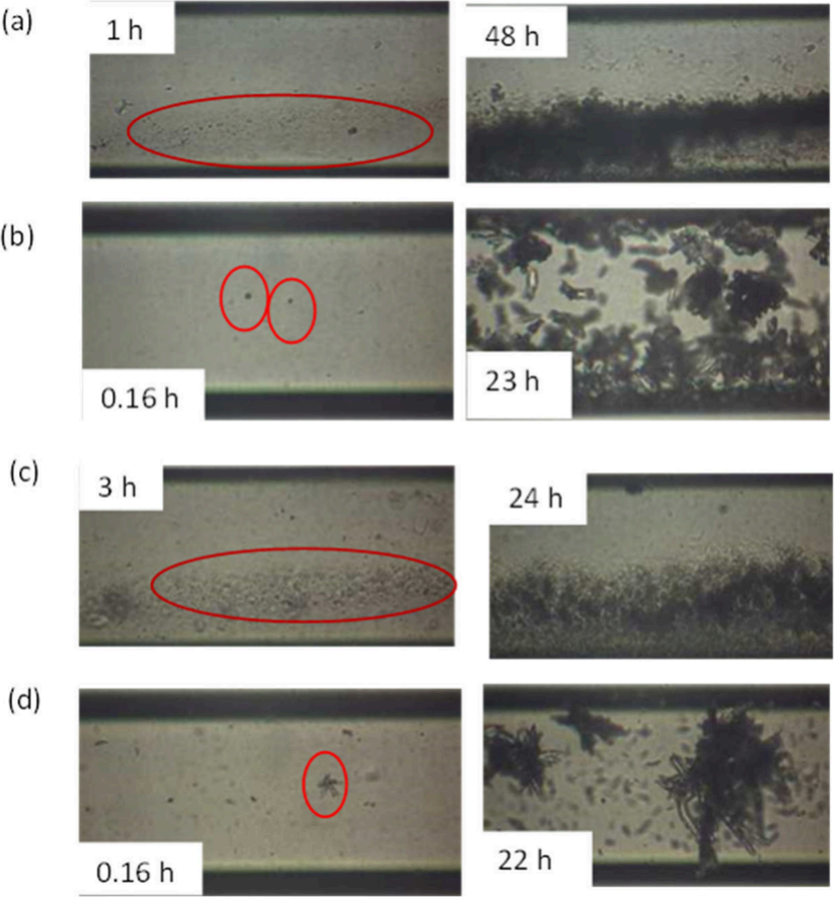
Snapshots of the first detected CaCO_3_ crystal in the presence of MEG (20% v/v) at (a) SR = 10.5 in the glass microchannel and (b) SR = 30.2 in the glass microchannel. (c) SR = 10.5 in the silane-coated microchannel. (d) SR = 30.2 in the silane-coated microchannel.

In the silane-coated microchannel at SR = 10.5 in the presence of MEG 20% v/v, the time of appearance of the first calcium carbonate crystal was ∼3 h, but similarly to the glass microchannel, ACC was formed (Figure 5(c)).

By increasing the SR value to 30.2, in the silane-coated microchannel, nucleation and crystal growth were accelerated. The first crystal of calcium carbonate was detected 0.16 h after the injection of the supersaturated solution. In the absence of MEG under similar experimental conditions, the first crystal was detected 3 h past mixing the CaCl_2_ and NaHCO_3_ solutions. The metastable polymorph aragonite was formed in the silane-coated microchannel in the presence of MEG 20% v/v.

During the flow and mixing of supersaturated solutions at SR = 10.5 containing MEG 20% v/v for both wettability conditions (glass microchannel and silane-coated microchannel), ACC was observed along the microchannels. In the case of the glass microchannel, increasing SR to 30.2, aragonite crystals and aggregates of aragonite crystals were detected along the entire length and clogged the microchip after 23 h of fluid flow (Figure S3). In the case of the silane-coated microchannel (SR = 30.2, MEG 20% v/v), crystal growth took place rather uniformly along the entire length of the microchip, which resulted in the formation of massive aragonite aggregates which clogged the microchip after 22 h of solution injection (Figure 6).

**Figure 6:**
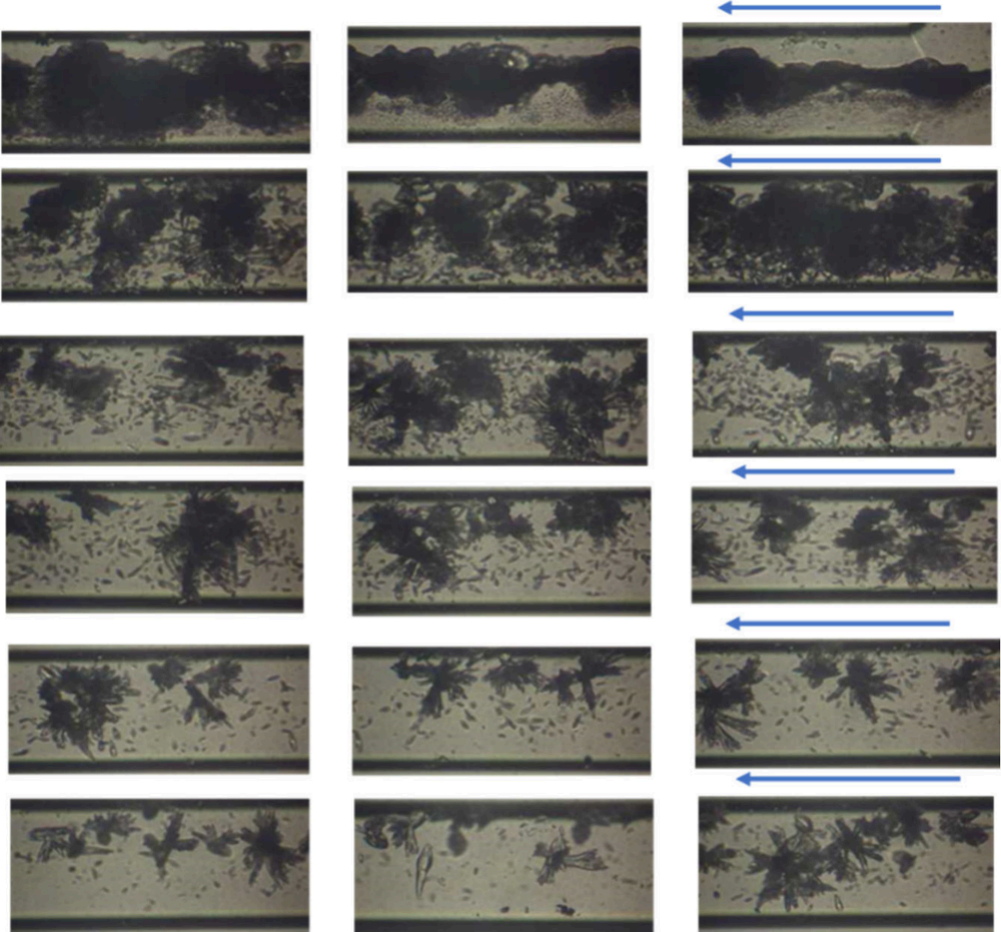
CaCO_3_ crystals along the silane-coated microchannel in the presence of MEG 20% v/v at SR = 30.2 (22 h of injection of solutions). Arrows show the flow direction.

Raman spectroscopy^25^ performed along the microchips after the experiments confirmed the characterization of CaCO_3_ polymorphs (Figure 7 and Table 3). The stabilization of polymorphs of calcium carbonate thermodynamically less stable than calcite may be attributed to the interaction of MEG on the surface of the growing supercritical nuclei and because of its effect on calcium carbonate solubility.^42–44^

**Figure 7:**
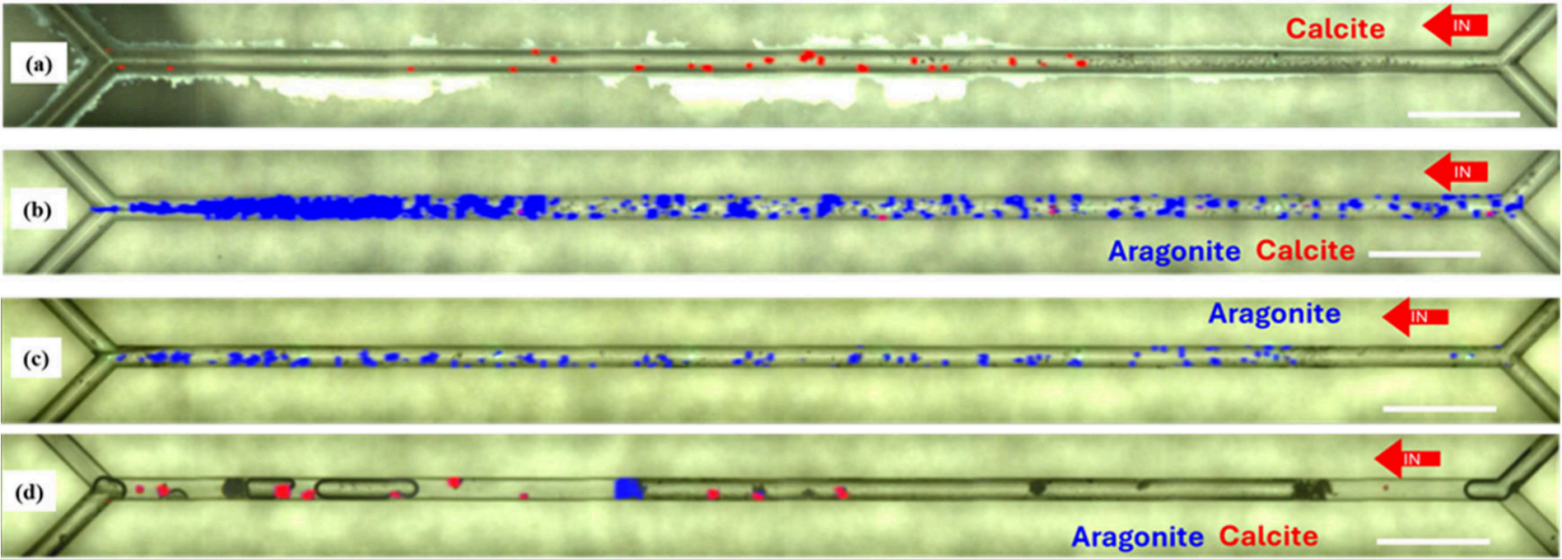
CaCO_3_ polymorphs identified using Raman spectroscopy; aragonite crystals are shown in blue, calcite in red, and amorphous calcium carbonate in gray. (a) Glass microchannel at SR = 10.5 and MEG 10% v/v (corresponding to Figure 3), (b) glass microchannel at SR = 30 and MEG 10% v/v (corresponding to Figure S1), (c) silane-coated microchannel at SR = 10.5 and MEG 10% v/v (corresponding to Figure S2), and (d) silane-coated microchannel at SR = 30 and 10% v/v MEG (corresponding to Figure 4).

**Table 3:** Precipitation Parameters of CaCO_3_ in Microchannels from Supersaturated Solutions^a^


SR	MEG (% v/v)	t_o_ (h)	R_c_ (μm/h)	DR_c_ %	CaCO_3_ Polymorphs
Glass Microchannel					
10.5	-	34.0 ± 0.0	3.3	-	Only calcite
10.5	10	5.5 ± 0.0	1.6	52	Calcite (ACC at short distances from mixing point) (Figure 7(a))
10.5	20	0.75 ± 0.35	-	100	Clouds of ACC and aragonite
30.2	-	24.0 ± 0.0	8.3	-	Calcite aggregates
30.2	10	1.25 ± 0.35	3.1	63	Aragonite and few calcite crystals (Figure 7(b))
30.2	20	0.18 ± 0.028	2.9	65	Aragonite prevails and few calcites
Silane-Coated Microchannel					
10.5	-	10.0 ± 0.0	(^a^)		Aragonite
10.5	10	6.25 ± 0.35	(^a^)		Aragonite aggregates (Figure 7(c))
10.5	20	2.75 ± 0.35	-	-	ACC
30.2	-	3.0 ± 000	8.6	-	Aragonite (vaterite close to the inlet)
30.2	10	0.5 ± 0.0	3.6	58.2	Calcite, aragonite and ACC (Figure 7(d))
30.2	20	0.18 ± 0.028	(^a^)	-	ACC and aragonite

a SR with respect to calcite, MEG concentration in the supersaturated solutions, mean time lapse for the observation of the first calcium carbonate crystal (t_o_) and standard deviation, crystal growth rate (R_c_), reduction of the rate of crystal growth (DR_c_), and crystal polymorphs formed.

b Very small aragonites did not allow the measurement of crystal size.

The snapshots obtained during the experiments allowed the measurement of the size of the first observed crystal as a function of time for the glass microchannel and silane-coated microchannel using ImageJ (Figure 8). When massive aragonite aggregates formed, it was impossible to measure the size of crystals for technical reasons, since aragonite aggregates present an irregular shape. For the obtained curves of crystal size as a function of time (Figure 8), calculations of the crystal growth rates were made using experimental data before the observation of the secondary nucleation (i.e., in the early stages of crystal growth) (Table 3).

**Figure 8:**
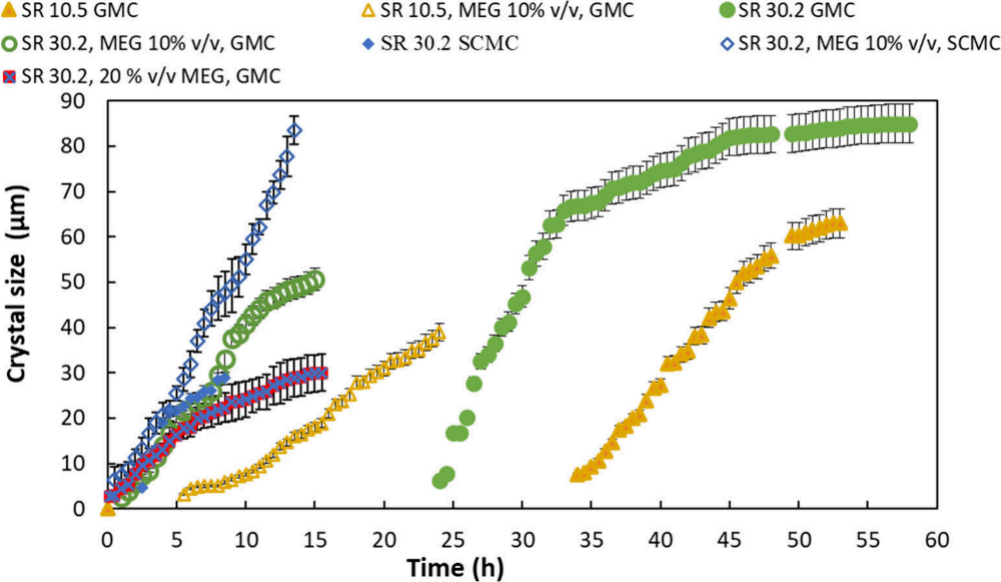
Crystal size of the first observed crystal as a function of time for the glass microchannel (GMC) and silane-coated microchannel (SCMC) in the presence (10% v/v, 20% v/v) and the absence of MEG for SR = 10.5 and 30.2.

The appearance of bursts of the massive formation of crystallite aggregates is apparently due to secondary nucleation. The crystal growth rates were calculated by using the crystal sizes before this stage. For both types of wettability, the presence of MEG at 10% v/v in the supersaturated solutions accelerated crystal formation and decreased the time for the detection of the first crystal formed in the respective supersaturated solutions and resulted in the formation of crystallites with smaller size in comparison to those formed in the absence of MEG. By increasing MEG’s concentration further, the time and size of the first observed crystal were both reduced. The crystal growth rate was decreased in the presence of MEG in the supersaturated solutions flowing in the glass microchips. However, in the silane-coated microchannel, the presence of aragonite crystallites and ACC did not allow the calculation of crystal growth rates except for the case of SR 30.2 and 10% MEG v/v in which a decrease was also observed due to MEG’s presence. In the presence of 20% v/v MEG, both glass and silane-coated glass microchips presented similar contact angle values with the supersaturated solutions; hence, their behavior with respect to calcium carbonate nucleation and growth was rather similar. The reduction % of the rate of crystal growth in the presence of MEG in the supersaturated solutions was calculated from eq 2:

2 $$\%\mathrm{rate reduction}=\frac{R_{0}-R_{\mathrm{MEG}}}{R_{0}}$$

In eq 2, R_0_ and R_MEG_ are the linear rates of crystal growth of calcium carbonate in the absence and in the presence of MEG, respectively.

For glass microchannels, the reduction in crystal growth rates reached 52% for SR = 10.5 in the presence of 10% MEG v/v, while the increase in MEG at 20% v/v reduced crystal growth 100%. The presence of 10% v/v MEG at SR = 30.2 reduced the growth rate by 63% for 20% v/v MEG, and the reduction was only 65%. This result was anticipated since inhibition is lower at higher supersaturation values. The crystal growth rate decrease in the presence of MEG is in good agreement with earlier reports.^17–19^ For the silane-coated microchannel reactors, the rate of crystal growth reduction could not be calculated since aragonite crystals and ACC formed. A high level of inhibition was reached for the high SR value of 30.2 for MEG concentration 10% v/v (i.e., the reduction of the crystal growth rate was 58.2%, and by further increasing the MEG’s concentration to 20% v/v the formation of aragonite crystals did not allow the crystal size measurements). In previous studies, a comparison of the times for observation of the first crystal, in glass and silane-coated microchips, showed that in silane-coated microchips, in the absence of MEG, local SR inhomogeneities due to the neutral wettability of the surface reduced the time needed for crystal observation. Herein, it is shown that the addition of 20% v/v MEG reduced this difference between the glass and silane-coated microchips.^25,28^ This quantitative information shows the effect of MEG’s presence during the mixing of the supersaturated solutions under flow conditions. Information on the early stages of crystal precipitation for specific fluid systems and solid surfaces was provided. When macroscopic mineral scaling takes place, it depends on the phenomena at the microscopic scale. Thus, the dependence of nucleation and crystal growth on the supersaturation of the solutions and in the presence or absence of other chemical substances with respect to the solid phase may be used for the prediction of macroscopic scale conditions.

## Conclusions

In this study, the effect of monoethylene glycol in calcium carbonate supersaturated solutions on calcium carbonate crystal formation was investigated. Direct visualization of CaCO_3_ crystals forming under flow conditions inside microchannels of varying wettability was performed. Two different supersaturation ratio (SR) values were tested (10.5 and 30.2) in the absence and in the presence of MEG at 10, 20, and 30% v/v.

The presence of 10% v/v of MEG in solutions supersaturated with respect to calcite, flowing in the microchannels at constant SR, shortened the time of appearance of the first crystal (induction time) and reduced the crystal growth rate. This effect was observed for both glass and silane-coated microchips which were, respectively, wet and neutral-wet by the supersaturated solutions. For both types of microchips, aragonite was stabilized in the presence of MEG while at high SR values aggregates of aragonite crystallites prevailed. In the absence of MEG, secondary nucleation was not significant in the glass microchips (wet by the used solutions); however, in the presence of MEG, secondary nucleation was noticeable.

Increasing MEG at 20% v/v altered the chemical affinity of the supersaturated solutions with the two types of microchips, and the wettability of both glass and silane-coated surfaces was neutral. At low SR values, the addition of MEG 20% v/v resulted in the formation of amorphous calcium carbonate (ACC) while increasing SR resulted in aragonite aggregates (i.e., the less stable polymorph), which is in close agreement with previous study.^16^ Increasing the MEG concentration in the supersaturated solutions to 30% v/v for both SR values resulted in complete inhibition of crystal formation. The presence of MEG decreased crystal growth rates for both the glass microchannel and silane-coated microchannel.

In this work, detailed observations were made on MEG’s effect on induction times and crystal growth rates of CaCO_3_ during the flow of very small liquid volumes and taking into consideration the chemical affinity of fluids with the channel walls. These results demonstrate the effect of MEG in calcium carbonate crystal formation and growth in the early stages of the precipitation process which should be considered for the control of calcium carbonate formation.

## Supplementary Material



## Notes

Additional experimental results (PDF)
