# Supplementary material for: Effect of Monoethylene Glycol on the Nucleation and Growth of Calcium Carbonate from Supersaturated Solutions in Microchannels of Varying Wettability
Source: Langmuir. 2025 May 16;41(21):13506–15. doi: 10.1021/acs.langmuir.5c01363 (PMC12139034; doi:10.1021/acs.langmuir.5c01363)
Supplement: Supplementary file 1 [file la5c01363_si_001.pdf]

SUPPLEMENTARY MATERIAL

Effect of Monoethylene Glycol on the Nucleation and Growth of Calcium Carbonate from Supersaturated Solutions in Microchannels of varying Wettability

Andreas Tzachristas[1,2], Dimitra Kanellopoulou[1,2], John Parthenios[2], Petros G. Koutsoukos[1,2], Christakis Paraskeva[1,2], Varvara Sygouni[*1,2]

[1]University of Patras/ Department of Chemical Engineering, Karatheodori 1, 26504 Patras, Greece

[2]Foundation for Research and Technology-Hellas/Institute of Chemical Engineering

*Corresponding author, tel: +30 2610 997574

E-mail address: sygouni@upatras.gr

CONTENTS

Figure S1. Snapshots of $CaCO_3$ crystal growth in the presence of MEG 10 % v/v along the glass microchannel at SR= 30.2

Figure S2. Snapshots of $CaCO_3$ crystal growth in the presence of MEG 10 % v/v along the silane coated microchannel at SR= 10.5

Figure S3. Snapshots of $CaCO_3$ crystal in the presence of MEG 20 % v/v along the glass microchannel at SR= 10.5

Figure S4. Snapshots of CaCO3 crystal growth in the presence of MEG 20 % v/v along the glass microchannel at SR= 10.5

Figure S5. A magnified area of Fig. 8 of the manuscript. Size of the first observed crystals as function of time.

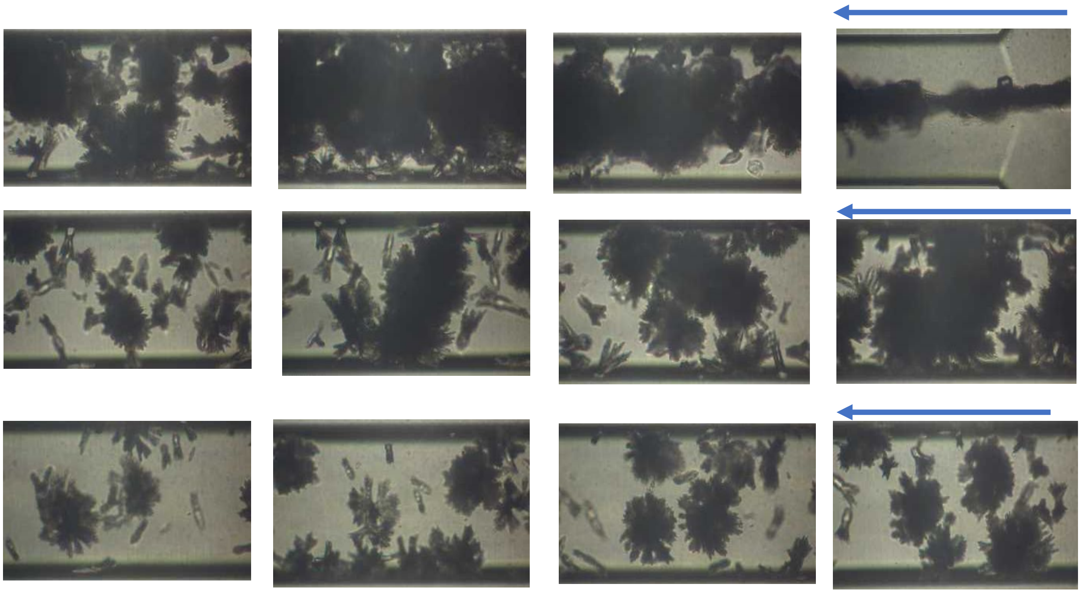

Figure S1. $CaCO_3$ crystal growth from solutions supersaturated with respect to calcium carbonate in the presence of MEG 10 % v/v along the glass microchannel at SR= 30.2 (24 h past the start of $CaCl_2$, $NaHCO_3$ solutions injection). Arrows show the flow direction.

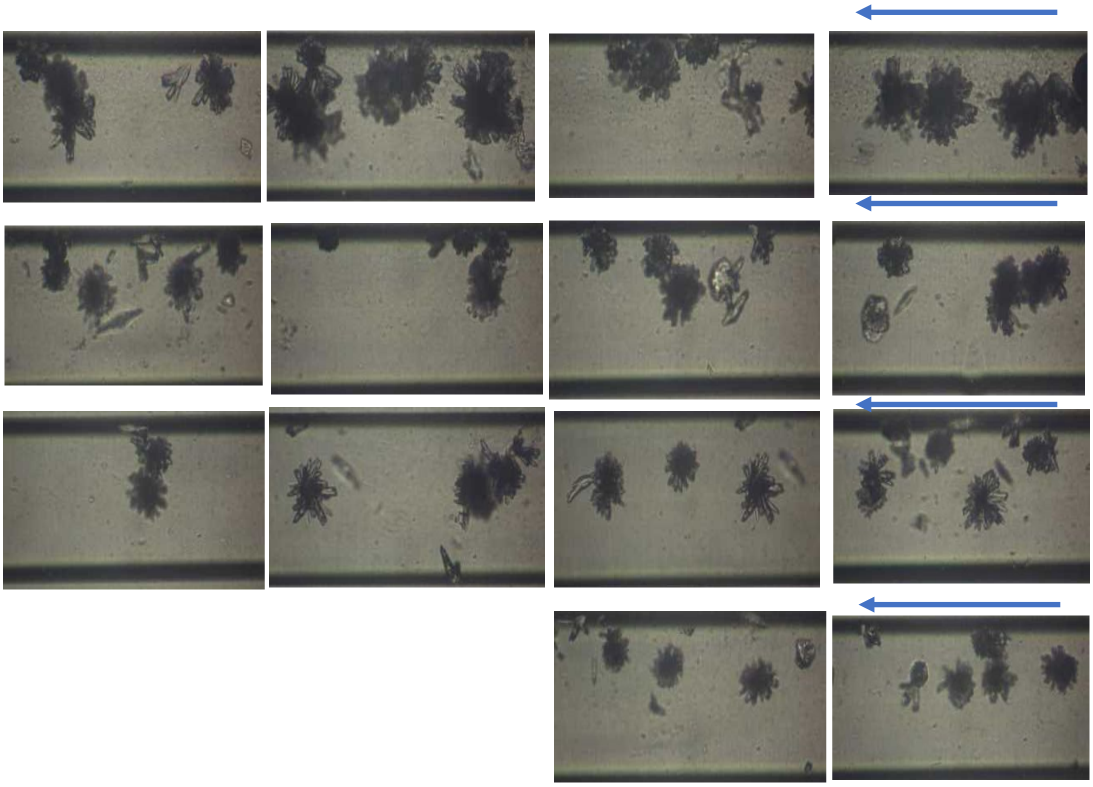

Figure S2. $CaCO_3$ crystal growth from solutions supersaturated with respect to calcium carbonate in the presence of MEG 10 % v/v along the silane coated microchannel at

SR= 10.5 (24 h past the start of $CaCl_2$, $NaHCO_3$ solutions injection). Arrows show the flow direction.

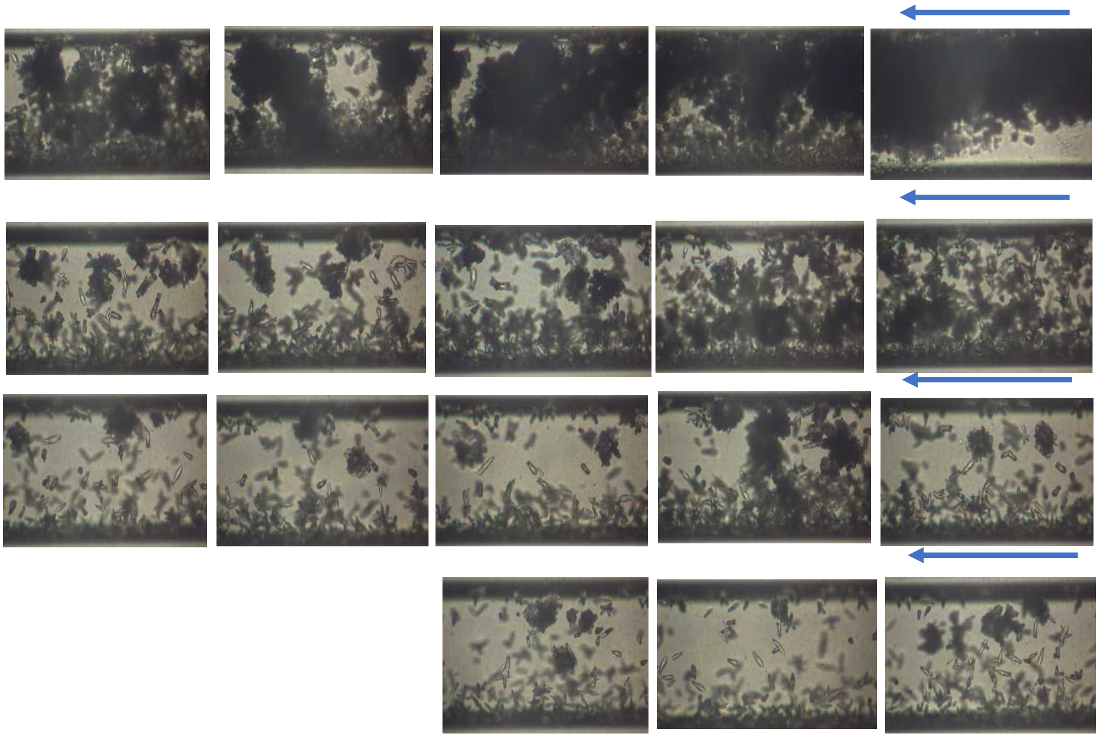

Figure S3. $CaCO_3$ crystal growth from solutions supersaturated with respect to calcium carbonate in the presence of MEG 20 % v/v along the glass microchannel at SR= 10.5 (23 h past the start of $CaCl_2$, $NaHCO_3$ solutions injection). Arrows show the flow direction.

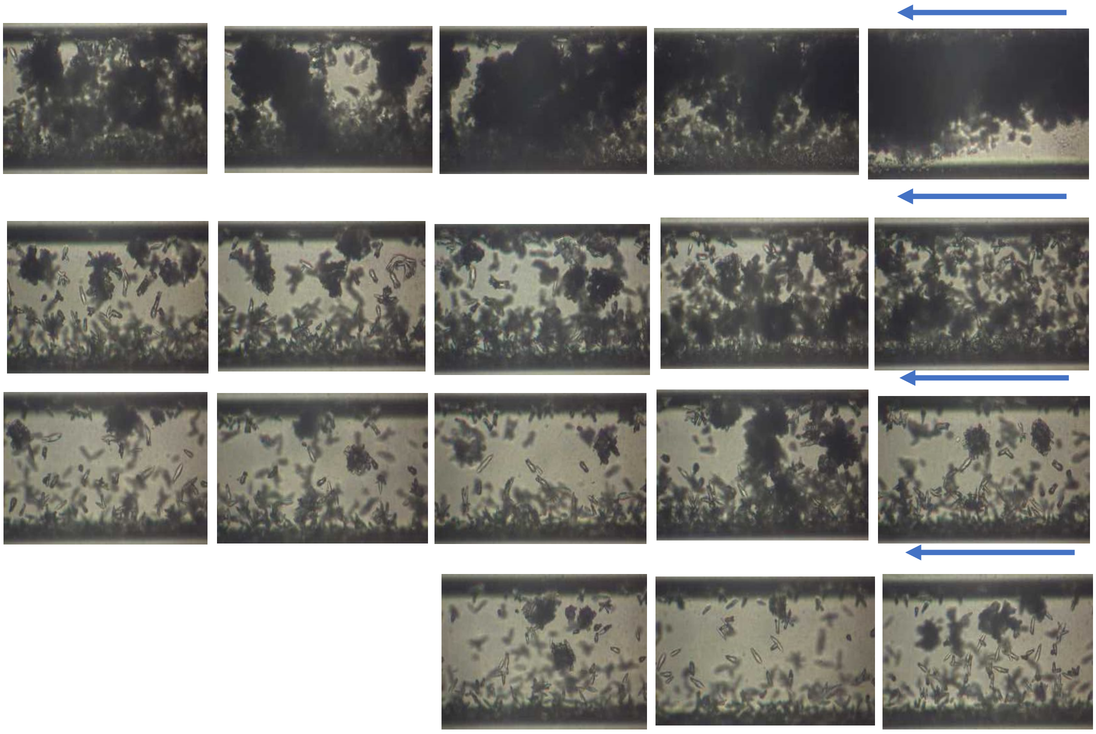

Figure S4. $CaCO_3$ crystal growth from solutions supersaturated with respect to calcium carbonate in the presence of MEG 20 % v/v along the glass microchannel at SR= 10.5 (23 h past the initiation of solutions injection). Arrows show the flow direction.

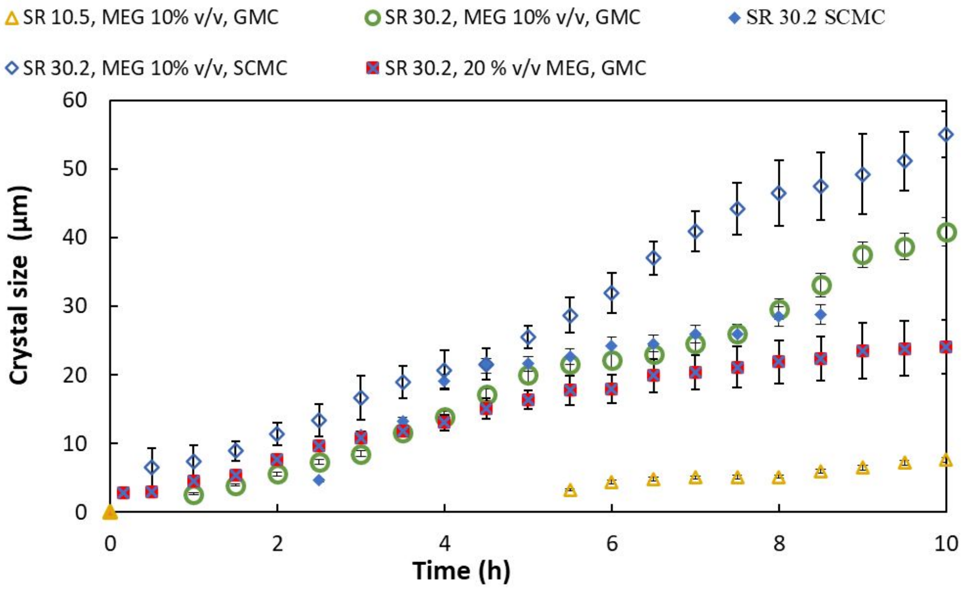

Figure S5. A magnified area of Fig. 8. Size of the first observed crystals as function of time for the glass microchannel (GMC) and silane coated microchannel (SCMC) in the presence (10 % v/v, 20 % v/v) and the absence of MEG for SR =10.5 and 30.2.
